# Tumor progression locus 2 reduces severe allergic airway inflammation by inhibiting Ccl24 production in dendritic cells

**DOI:** 10.1016/j.jaci.2016.05.031

**Published:** 2017-02

**Authors:** Yashaswini Kannan, Yanda Li, Stephanie M. Coomes, Isobel S. Okoye, Victoria S. Pelly, Srividya Sriskantharajah, Eva Gückel, Lauren Webb, Stephanie Czieso, Nikolay Nikolov, Andrew S. MacDonald, Steven C. Ley, Mark S. Wilson

**Affiliations:** a Francis Crick Institute, Mill Hill Laboratory, the Ridgeway, London, United Kingdom; b Faculty of Life Sciences, Manchester Collaborative Centre for Inflammation Research, the University of Manchester, Manchester, United Kingdom

**Keywords:** TPL-2, *Map3k8*^*−*/*−*^, allergy, severe asthma, house dust mite, dendritic cells, Ccl24, eotaxin-2, BAL, Bronchoalveolar lavage, BM, Bone marrow, BMDC, Bone marrow–derived dendritic cell, DC, Dendritic cell, ERK, Extracellular-signal regulated kinase, HDM, House dust mite, i.n., Intranasal, LN, Lymph node, medLN, Mediastinal lymph node, TCR, T-cell receptor, TLR, Toll-like receptor, TPL-2, Tumor progression locus 2, WT, Wild-type

## Abstract

**Background:**

The molecular and cellular pathways driving the pathogenesis of severe asthma are poorly defined. Tumor progression locus 2 (TPL-2) (COT, MAP3K8) kinase activates the MEK1/2-extracellular-signal regulated kinase 1/2 MAP kinase signaling pathway following Toll-like receptor, TNFR1, and IL-1R stimulation.

**Objective:**

TPL-2 has been widely described as a critical regulator of inflammation, and we sought to investigate the role of TPL-2 in house dust mite (HDM)-mediated allergic airway inflammation.

**Methods:**

A comparative analysis of wild-type and *Map3k8*^*−*/*−*^ mice was conducted. Mixed bone marrow chimeras, conditional knockout mice, and adoptive transfer models were also used. Differential cell counts were performed on the bronchoalveolar lavage fluid, followed by histological analysis of lung sections. Flow cytometry and quantitative PCR was used to measure type 2 cytokines. ELISA was used to assess the production of IgE, type 2 cytokines, and Ccl24. RNA sequencing was used to characterize dendritic cell (DC) transcripts.

**Results:**

TPL-2 deficiency led to exacerbated HDM-induced airway allergy, with increased airway and tissue eosinophilia, lung inflammation, and IL-4, IL-5, IL-13, and IgE production. Increased airway allergic responses in *Map3k8*^*−*/*−*^ mice were not due to a cell-intrinsic role for TPL-2 in T cells, B cells, or LysM^+^ cells but due to a regulatory role for TPL-2 in DCs. TPL-2 inhibited *Ccl24* expression in lung DCs, and blockade of Ccl24 prevented the exaggerated airway eosinophilia and lung inflammation in mice given HDM-pulsed *Map3k8*^*−*/*−*^ DCs.

**Conclusions:**

TPL-2 regulates DC-derived Ccl24 production to prevent severe type 2 airway allergy in mice.

Allergic asthma is a hyperinflammatory disease of the airways, initiated in atopic individuals by allergen-presenting dendritic cells (DCs) leading to the recruitment of cytokine-secreting T cells and eosinophils to the lungs.^1^, ^2^, ^3^ Repeated allergen exposure results in local tissue inflammation and remodeling with systemic elevated levels of circulating IgE.^4^ Despite a good understanding of the pathogenesis of allergic asthma, the mainstay for treatment continues to be inhaled broad-scale corticosteroids, which have a number of significant side effects. Furthermore, an increasing number of patients are resistant to corticosteroid treatment^5^ and there are an increasing number of patients who develop severe asthma. Consequently, there is an urgent medical need to understand in more detail the molecular mechanisms of severe allergic asthma to identify new therapeutic targets.^6^, ^7^

The mitogen activated protein-3 kinase tumor progression locus 2 (TPL-2; also known as mitogen-activated protein kinase kinase kinase 8 [MAP3K8] and cancer Osaka thyroid oncogene [COT]) phosphorylates and activates MEK1/2 after stimulation of Toll-like receptors (TLRs) and the receptors for TNF and IL-1β, leading to the activation of extracellular-signal regulated kinase (ERK) 1/2 MAP kinases.^8^ The TPL-2/ERK1/2 signaling pathway regulates cytokine and chemokine production in inflammatory responses.^9^, ^10^, ^11^ Studies with TPL-2–deficient *Map3k8*^*−*/*−*^ mice have indicated that TPL-2 promotes inflammation in models of endotoxin shock, pancreatitis, liver fibrosis, and thrombocytopenia.^9^, ^12^, ^13^, ^14^ TPL-2 is also required for proficient immunity to intracellular bacterial and protozoan infection.^15^, ^16^

We, and others, demonstrated that TPL-2 signaling in radiation-resistant stromal cells, but not T cells or any other hematopoietic cell, promotes the onset and severity of experimental autoimmune encephalomyelitis, a model of multiple sclerosis.^17^, ^18^ Although these studies highlight the importance of the TPL-2/MEK/ERK signaling axis in type 1 and T_H_17 immune responses, the role of TPL-2 in mediating type 2 responses has not been clearly established. A previous study suggested that T-cell–intrinsic TPL-2 regulated CD4^+^ T_H_2 cell differentiation *in vitro* via ERK1/2 activation.^19^ The authors subsequently hypothesized that increased type 2–associated ovalbumin-induced airway inflammation in TPL-2–deficient mice was due to a T-cell–intrinsic deficiency of TPL-2; however, this was not tested. In our studies, we found that T-cell receptor (TCR) activation of ERK1/2 in purified CD4^+^ T cells was completely independent of TPL-2.^17^ These results prompted us to formally test whether T-cell–intrinsic TPL-2 was required for type 2 immunity *in vivo* using a clinically relevant allergen, house dust mite (HDM),^20^ in various models of allergic airway inflammation.

In the present study, we show that TPL-2 deficiency led to severe HDM-induced airway allergy, when compared with wild-type (WT) HDM-treated mice. Using adoptive transfer experiments and cell lineage–specific conditional knockout mice, we show that TPL-2 in T cells and B cells was not required for control of severe airway allergy after HDM challenge. Rather, we found an essential role for TPL-2 in DCs, restraining their promotion of excessive airway inflammation. Using several *in vivo* models with genomewide RNA sequencing, we identified that TPL-2 regulated the expression and production of Ccl24 (eotaxin-2) by DCs. Furthermore, blocking Ccl24 *in vivo* abrogated the exacerbated airway inflammation induced by TPL-2–deficient DCs, demonstrating a previously unappreciated role for DC-intrinsic TPL-2 in regulating Ccl24 to limit severe airway allergy.

## Methods

For detailed Methods, see this article's Online Repository at www.jacionline.org.

## Results

### TPL-2 inhibits HDM-induced airway allergy

**Fig E1:**
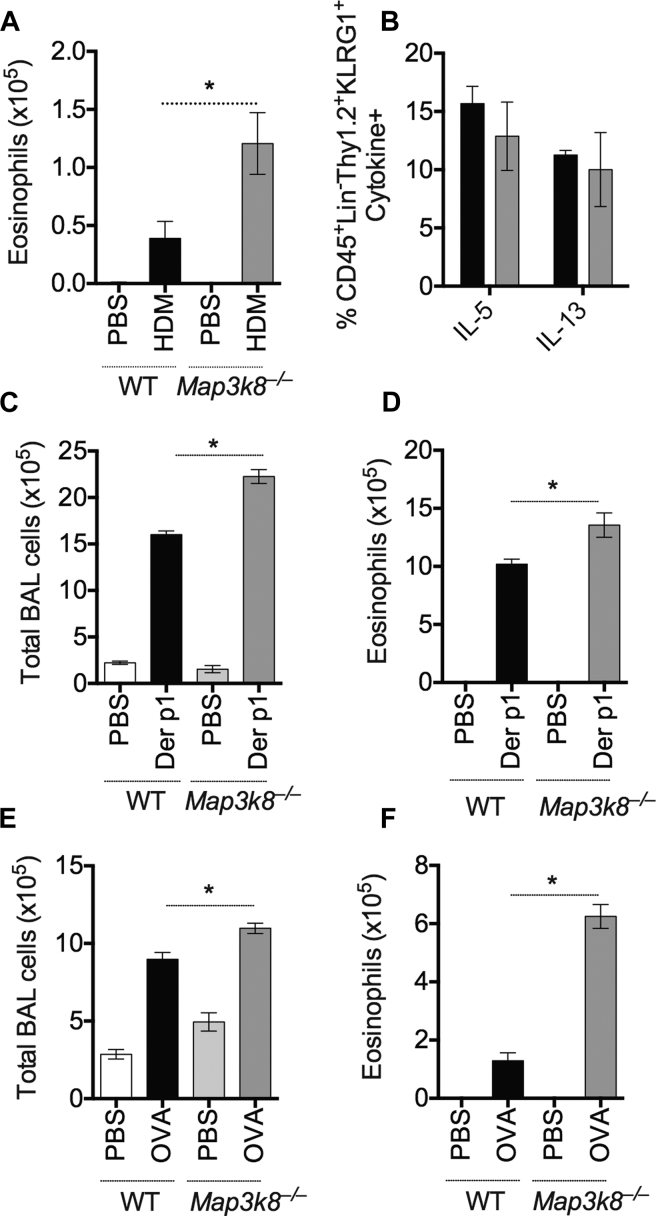
Allergic airway responses in WT and *Map3k8*^*−*/*−*^ mice. **A,** Total number of lung eosinophils (SiglecF^+^/CD11c^−^) in PBS-treated and allergic WT and *Map3k8*^*−*/*−*^ mice as assessed by ICS. **B,** Frequency of IL-5^+^ and IL-13^+^ Lin-/Thy1.2^+^/KLRG1^+^ group 2 innate lymphoid cells in the allergic lungs of WT and *Map3k8*^*−*/*−*^ mice as assessed by ICS. **C** and **D,** Total and eosinophilic counts in the BAL fluid of WT and *Map3k8*^*−*/*−*^ mice sensitized with alum and *Der p1* via the i.p. route and challenged with *Der p1* intratracheally. **E** and **F,** Total and eosinophilic counts in the BAL fluid of WT and *Map3k8*^*−*/*−*^ mice sensitized with alum and OVA via the i.p. route and challenged with OVA intratracheally. All experiments are representative of 2 to 3 independent experiments with 4 to 5 mice/genotype. *ICS*, Intracellular cytokine staining; *i.p*., intraperitoneal; OVA, ovalbumin. *P < .05 as assessed by the 2-tailed Mann-Whitney test.

**Fig 1:**
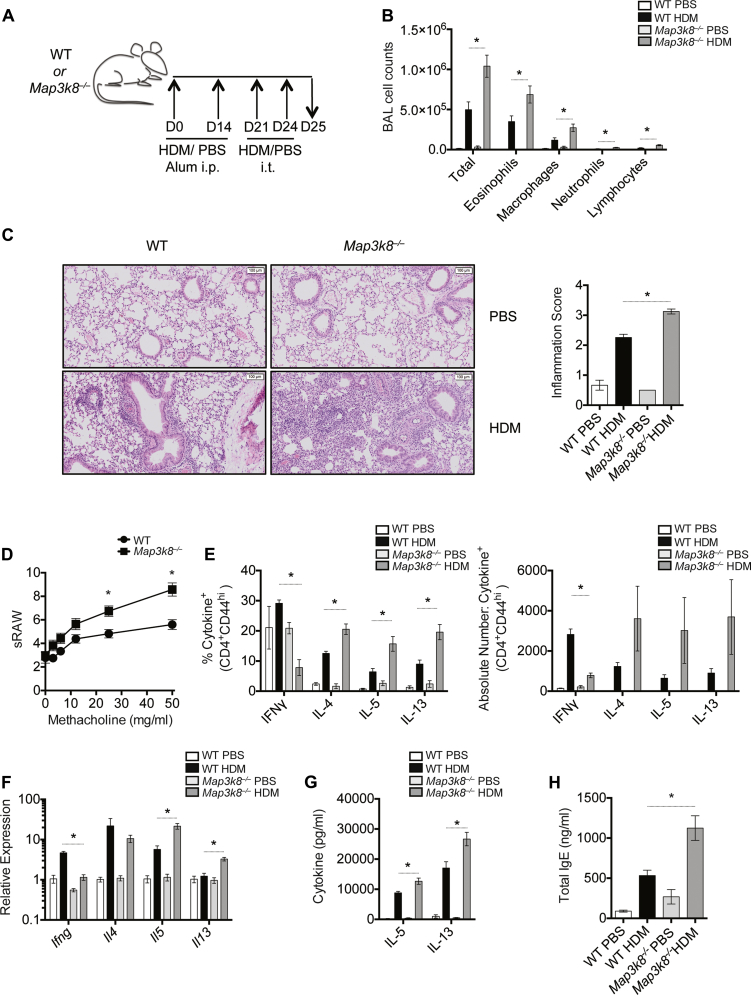
*Map3k8*^*−*/*−*^ mice mount enhanced airway allergic responses compared with WT mice. **A,** Schematic representation of the i.p. alum-based HDM sensitization and i.t. HDM challenge in WT and *Map3k8*^*−*/*−*^ mice. **B,** Differential counts in the BAL fluid of PBS- and HDM-challenged WT and *Map3k8*^*−*/*−*^ mice. **C,** Hematoxylin and eosin *(H&E)*-stained histology sections and inflammation scores from PBS- and HDM-challenged lungs of WT and *Map3k8*^*−*/*−*^ mice depicting cellular infiltration. **D,** Airway resistance (sRAW) measurement in allergic WT and *Map3k8*^*−*/*−*^ mice in response to increasing doses of methacholine (3-50 mg/mL). **E,** Frequency and total number of cytokine^+^/CD4^+^/CD44^hi^ cells in the lungs of PBS- and HDM-challenged WT and *Map3k8*^*−*/*−*^ mice as assessed by intracellular cytokine staining. **F,** Lung expression of *Ifng*, *Il4*, *Il5*, and *Il13* in PBS- and HDM-challenged WT and *Map3k8*^*−*/*−*^ mice. **G,** HDM-specific IL-5 and IL-13 protein production as assessed by ELISA in the medLN cell culture supernatants from PBS- and HDM-challenged WT and *Map3k8*^*−*/*−*^ mice for 4 days. **H,** Total IgE in the serum as assessed by ELISA from PBS- and HDM-challenged WT and *Map3k8*^*−*/*−*^ mice. All experiments are representative of 2 to 3 independent experiments with 4 to 5 mice/genotype. The airway resistance response is data combined from 2 independent experiments with n = 4 mice/group. *P < .05 as assessed by the 2-tailed Mann-Whitney test. *i.p*., Intraperitoneal; *i.t*., intratracheal.

Intraperitoneal allergen sensitization followed by localized airway challenge is a well-established CD4^+^ T-cell–dependent model of airway allergy.^21^ To investigate the role of TPL-2 in airway allergy, we sensitized and challenged WT and *Map3k8*^*−*/*−*^ mice with HDM, one of the most common aeroallergens affecting humans^20^ (Fig 1, *A*). One day after the final intratracheal airway challenge, we observed significantly increased total cellular infiltration in the bronchoalveolar lavage (BAL) fluid of *Map3k8*^*−*/*−*^ mice compared with WT mice (Fig 1, *B*). We observed no baseline differences between the 2 groups of mice after PBS injection (Fig 1). Differential cell counts revealed that *Map3k8*^*−*/*−*^ mice had significantly increased numbers of eosinophils, macrophages, neutrophils, and lymphocytes in the BAL fluid (Fig 1, *B*). In addition, *Map3k8*^*−*/*−*^ mice had significantly increased numbers of eosinophils in the lung compared with WT mice (see Fig E1, *A*, in this article's Online Repository at www.jacionline.org). Accompanying the increased airway inflammation, we also observed augmented perivascular and peribronchial tissue inflammation and a loss of lung architecture in TPL-2–deficient mice (Fig 1, *C*). Consistent with the increased pulmonary inflammation, we also observed a significant increase in airway resistance in HDM-challenged *Map3k8*^*−*/*−*^ mice upon administration of increasing doses of methacholine compared with HDM-challenged WT mice (Fig 1, *D*).

In line with elevated airway and tissue inflammation in *Map3k8*^*−*/*−*^ mice, we observed an increased frequency and total number of IL-4–, IL-5–, and IL-13–secreting T_H_2 cells, with decreased IFN-γ–secreting T cells in the lungs of *Map3k8*^*−*/*−*^ mice compared with WT controls (Fig 1, *E*). The frequency of IL-5– and IL-13–secreting group 2 innate lymphoid cells was unchanged (Fig E1, *B*). Consistent with elevated lung T_H_2 cells, *Map3k8*^*−*/*−*^ mice had significantly increased mRNA expression for type 2 cytokines *Il5* and *Il13* in the lung (Fig 1, *F*). Although the expression of *Ifng* was significantly reduced, that of *Il4* remained unchanged in *Map3k8*^*−*/*−*^ mice compared with WT controls (Fig 1, *F*). In accordance with this, HDM-specific recall responses in the local draining lymph node (LN) cultures revealed increased IL-5 and IL-13 production by *Map3k8*^*−*/*−*^ mediastinal lymph node (medLN) cells (Fig 1, *G*). Serum levels of IgE were also elevated in *Map3k8*^*−*/*−*^ mice compared with WT mice (Fig 1, *H*).

Activation of the TLR4 signaling pathway contributes significantly to airway allergy after HDM challenge.^22^, ^23^ Given that TPL-2 signals downstream of TLR4, we tested whether the observed increased airway allergy in *Map3k8*^*−*/*−*^ mice was restricted to HDM-induced allergy. Sensitization and airway challenge with *Der p1*, the cysteine protease component of HDM or chicken egg ovalbumin, also invoked significantly increased allergic airway inflammation in *Map3k8*^*−*/*−*^ mice, compared with WT mice (Fig E1, *C*-*F*). Together, these data extend the previous report^19^ and clearly demonstrate an important regulatory role for TPL-2 in modulating allergic airway responses in several model systems.

### Neither T-cell–intrinsic nor B-cell–intrinsic TPL-2 regulates HDM-induced airway allergy

Although it was previously suggested that T-cell–intrinsic TPL-2 was responsible for elevated airway inflammation in TPL-2–deficient mice, this was not directly tested *in vivo*.^19^ We therefore assessed whether TPL-2 function specifically in T cells was responsible for the increased allergic airway responses in complete *Map3k8*^*−*/*−*^ mice, using 2 independent and complementary approaches.

**Fig 2:**
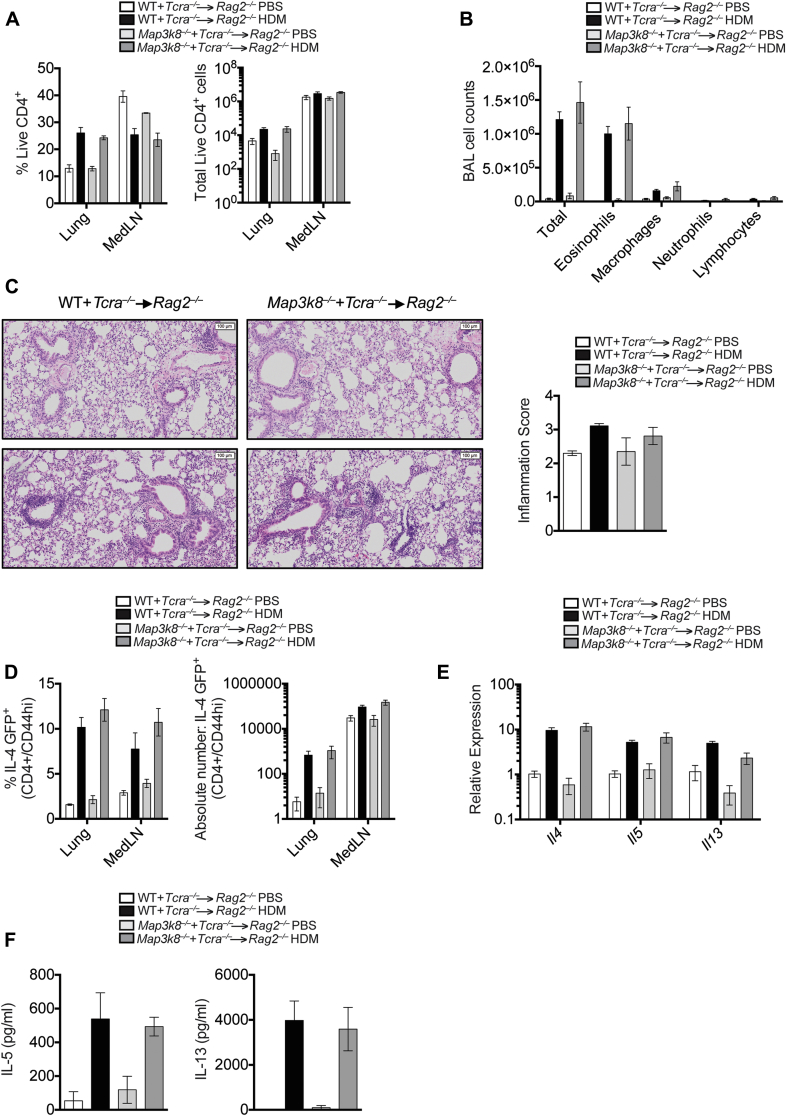
Increased airway allergic responses in *Map3k8*^*−*/*−*^ mice is not a result of T-cell–intrinsic TPL-2 function. **A,** CD4 T-cell reconstitution in the lung and medLNs of PBS- and HDM-challenged chimeric mice. *White bars* represent PBS-challenged chimeric mice with WT T cells, *black bars* represent HDM-challenged chimeric mice with WT T cells, *light gray bars* represent PBS-challenged chimeric mice with *Map3k8*^*−*/*−*^ T cells, and *dark gray bars* represent HDM-challenged chimeric mice with *Map3k8*^*−*/*−*^ T cells. **B,** Differential counts in the BAL fluid of PBS- and HDM-challenged chimeric mice. **C,** Hematoxylin and eosin–stained lung histology sections and inflammation scores from PBS-challenged and allergic chimeric mice. **D,** Frequency and total number of IL-4GFP^+^/CD4^+^/CD44^hi^ cells in the lungs of PBS- and HDM-challenged chimeric mice. **E,** Lung expression of *Il4*, *Il5*, and *Il13* mRNA in PBS- and HDM-challenged chimeric mice. **F,** HDM-specific IL-5 and IL-13 protein production as assessed by ELISA in the medLN cell culture supernatants from PBS- and HDM-challenged chimeric mice for 4 days. All experiments are representative of 3 independent experiments with 4 to 5 mice/genotype.

First, we established mixed bone marrow (BM) chimeric mice with 80% *Tcra*^*−*/*−*^ BM cells mixed with 20% WT or 20% *Map3k8*^*−*/*−*^ BM cells transferred to *Rag2*^*−*/*−*^ hosts. In the resulting chimeras, all the T cells developed from either WT or TPL-2–deficient donor BM, whereas the majority (80%) of other hematopoietic cells were derived from TPL-2–sufficient *Tcra*^*−*/*−*^ BM. Reconstitution of CD4^+^ T cells between the 2 chimeric groups was identical (Fig 2, *A*). Following HDM sensitization and airway challenge, we observed no differences in the total cellular infiltration or the number of eosinophils in the BAL fluid of chimeric mice with WT T cells or TPL-2–deficient T cells (Fig 2, *B*). Similarly, pulmonary inflammation was comparable between the 2 groups of chimeric mice (Fig 2, *C*). BM from WT *Il4*^*GFP*+^ and *Map3k8*^*−*/*−*^ *Il4*^*GFP*+^ mice was used to reconstitute the chimeric mice, allowing us to track T_H_2 cells in the chimeric mice by flow cytometry. We performed fluorescence-activated cell sorting analysis on the lung and LN cell populations and observed no significant differences in the frequency and number of CD4^+^CD44^hi^*Il4*^*GFP*+^ cells (Fig 2*, D*). Furthermore, no differences were detected in the expression of *Il4, Il5*, and *Il13* in the lungs of allergen-challenged chimeric mice (Fig 2, *E*). Finally, HDM restimulation of medLN cells from each group of chimeric mice resulted in similar levels of IL-5 and IL-13 secretion (Fig 2, *F*).

**Fig E2:**
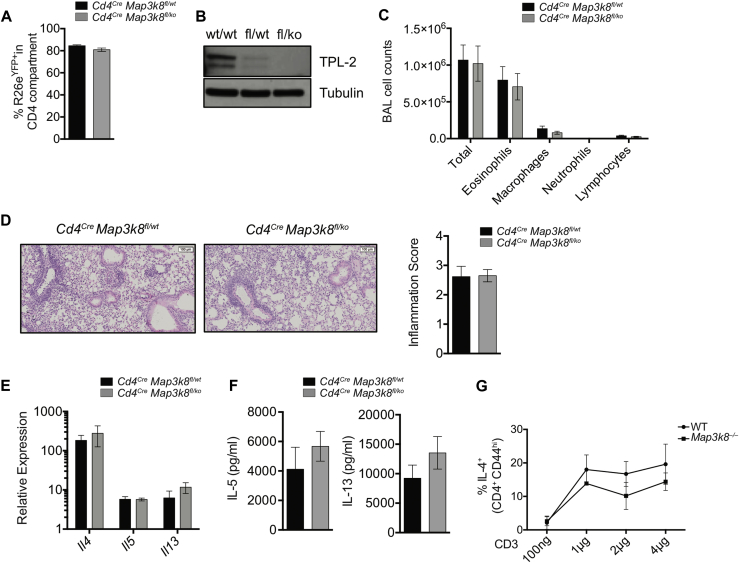
Role of TPL-2 function in CD4^+^ cells upon HDM sensitization and challenge. **A,** Frequency of R26e^YFP+^ in the CD4^+^ T-cell compartment from lungs of allergic conditional KO mice. *Black bars* represent *Cd4*^*Cre*^*Map3k8*^*fl/wt*^ mice, and *gray bars* represent *Cd4*^*Cre*^*Map3k8*^*fl/ko*^ mice. **B,** Protein expression of TPL-2 (Santa Cruz Biotechnology, Dallas, Tex) and tubulin in splenic CD4^+^/R26e^YFP^^+^ T cells from conditional KO mice assessed by western blotting. For the WT cells, CD4^+^ T cells were isolated as control. **C,** Differential counts in the BAL fluid of allergic *Cd4*^*Cre*^ conditional KO mice. **D,** Hematoxylin and eosin–stained histology sections and inflammation scores from allergic lungs of *Cd4*^*Cre*^ conditional KO mice. **E,** Lung expression of *Il4*, *Il5*, and *Il13* mRNA in allergic *Cd4*^*Cre*^ conditional KO mice. **F,** HDM-specific IL-5 and IL-13 protein production as assessed by ELISA in the culture supernatants of medLN cells from allergic *Cd4*^*Cre*^ conditional KO mice for 4 days. **G,** Frequency of IL-4^+^/CD4^+^/CD44^hi^ cells in 6d *in vitro* polarized T_H_2 cultures of naive T cells isolated from WT and *Map3k8*^*−*/*−*^ mice as assessed by intracellular cytokine staining. All experiments are representative of 3 independent experiments with 4 to 6 mice/genotype. *KO*, Knockout.

Second, we used a Cre expression system under the *Cd4* promoter to delete TPL-2 in T cells using mice with a LoxP-flanked *Map3k8* allele. Concurrent deletion of a LoxP-flanked stop codon ahead of the eYFP reporter under the *Rosa26* promoter facilitated evaluation of deletion efficiency. We sensitized and challenged mice with TPL-2–sufficient (*Cd4*^*Cre*^*Map3k8*^*fl/wt*^) or TPL-2–deficient (*Cd4*^*Cre*^*Map3k8*^*fl/ko*^) CD4^+^ cells (see Fig E2, *A* and *B*, in this article's Online Repository at www.jacionline.org). In accordance with the results obtained with chimeric mice, we found no differences in the total cellular infiltration in the BAL fluid or the number of eosinophils between *Cd4*^*Cre*^*Map3k8*^*fl/ko*^ mice and control *Cd4*^*Cre*^*Map3k8*^*fl/wt*^ mice (Fig E2, *C*). Pulmonary inflammation was also comparable between the genotypes (Fig E2, *D*), with no difference in mRNA expression for type 2 cytokines in the lung (Fig E2, *E*) or the secretion of IL-5 and IL-13 by HDM-restimulated medLN cells (Fig E2, *F*). Together, these complementary experiments clearly demonstrated that the enhanced allergic responses to HDM in *Map3k8*^*−*/*−*^ mice did not result from the absence of TPL-2 expression in T cells alone. Consistent with this conclusion, TPL-2–deficient T cells were comparable to WT T cells in their ability to differentiate to the T_H_2 lineage and produce IL-4 *in vitro* (Fig E2, *G*).

**Fig E3:**
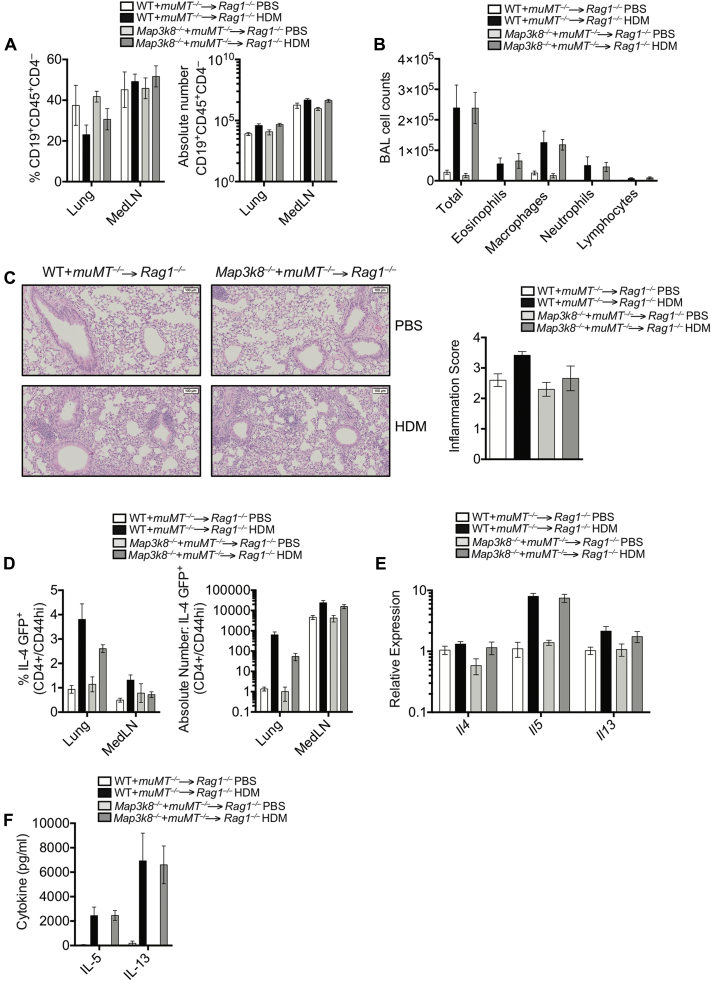
Severe allergic responses in *Map3k8*^*−*/*−*^ mice independent of TPL-2 function in B cells. **A,** CD19^+^ B-cell reconstitution in the lung and medLNs of PBS- and HDM-challenged chimeric mice. *White bars* represent PBS-challenged chimeric mice with WT B cells, *black bars* represent HDM-challenged chimeric mice with WT B cells, *light gray bars* represent PBS-challenged chimeric mice with *Map3k8*^*−*/*−*^ B cells, and *dark gray bars* represent HDM-challenged chimeric mice with *Map3k8*^*−*/*−*^ B cells. **B,** Differential counts in the BAL fluid of PBS- and HDM-challenged chimeric mice. **C**, H&E-stained lung histology sections and inflammation scores from PBS-challenged and allergic chimeric mice. **D,** Frequency and total number of IL-4GFP^+^/CD4^+^/CD44^hi^ cells in the lungs of PBS- and HDM-challenged chimeric mice. **E**, Lung expression of *Il4*, *Il5*, and *Il13* mRNA in PBS- and HDM-challenged chimeric mice. **F,** HDM-specific IL-5 and IL-13 protein production as assessed by ELISA in the medLN cell culture supernatants from PBS- and HDM-challenged chimeric mice for 4 days. All experiments are representative of 2 independent experiments with 4 to 5 mice/genotype.

Because various B-cell populations can regulate airway allergy,^24^ we next tested the role of B-cell–intrinsic TPL-2, using a similar mixed BM chimeric system with 80% *mu*MT BM mixed with 20% WT or 20% *Map3k8*^*−*/*−*^ BM cells transferred to *Rag1*^*−*/*−*^ hosts. The resulting chimeric mice had similar reconstitution (see Fig E3, *A*, in this article's Online Repository at www.jacionline.org) and upon HDM sensitization and airway challenge mounted a comparable airway inflammatory response, with similar airway infiltrates (Fig E3, *B*), comparable pulmonary inflammation (Fig E3, *C*), similar expression of *ll4*^*GFP*^ in CD4^+^ cells in lung and LN (Fig E3, *D*), lung mRNA expression for type 2 cytokines (Fig E3, *E*), and HDM-specific cytokine secretion by restimulated medLN cells (Fig E3, *F*). Together, these observations indicated that the exacerbated allergic airway inflammation observed in TPL-2–deficient mice was not due to the absence of TPL-2 in the T-cell or B-cell compartments.

### TPL-2 function in CD11c^+^ DCs regulates HDM-induced airway allergy

**Fig 3:**
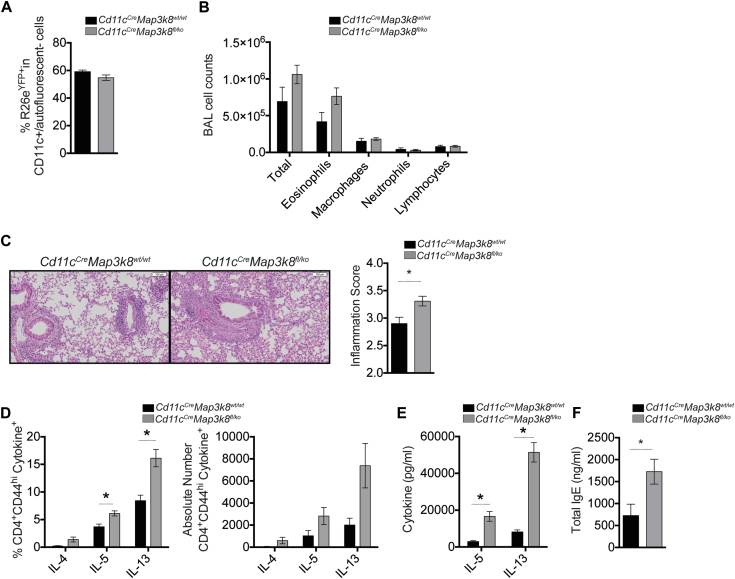
*Map3k8*^*−*/*−*^ mice mount increased allergic responses due to TPL-2 function in CD11c+ DCs. **A,** Frequency of R26e^YFP+^ in the CD11c^+^ DCs from lungs of allergic conditional KO mice. *Black bars* represent *Cd11c*^*Cre*^*Map3k8*^*wt/wt*^ mice, and *gray bars* represent *Cd11c*^*Cre*^*Map3k8*^*fl/ko*^ mice. **B,** Differential counts in the BAL fluid of allergic conditional KO mice. **C,** Hematoxylin and eosin–stained histology sections and inflammation scores from allergic lungs of conditional KO mice showing increased cellular infiltration. **D,** Frequency and total number of cytokine^+^/CD4^+^/CD44^hi^ cells in the allergic lungs of conditional KO mice as assessed by intracellular cytokine staining. **E,** HDM-specific IL-5 and IL-13 protein production as assessed by ELISA in the medLN cell culture supernatants from allergic conditional KO mice for 4 days. **F,** Total IgE in the serum as assessed by ELISA from allergic conditional KO mice. All experiments are representative of 2 independent experiments with 3 to 5 mice/genotype. *P < .05 as assessed by Mann-Whitney test. *KO*, Knockout.

TPL-2 has well-established signaling functions in innate immune cells,^8^, ^10^ including DCs, which are required for allergen-induced airway inflammation.^25^ To test whether TPL-2 in CD11c^+^ DCs regulated allergic airway responses, we restricted the deletion of TPL-2 to CD11c^+^ cells using *Cd11c*^*Cre*^*Map3k8*^*fl/ko*^ mice and used the expression of the eYFP reporter to evaluate the efficiency of deletion (Fig 3, *A*). Following HDM sensitization and challenge of *Cd11c*^*Cre*^*Map3k8*^*fl/ko*^ mice, we observed an increase in the influx of total cells and eosinophils in the BAL fluid, compared with control *Cd11c*^*Cre*^*Map3k8*^*wt/wt*^ mice; however, this change was not statistically significant (Fig 3, *B*). This trend was accompanied by significantly increased inflammation in the lungs, with perivascular and peribronchial cellular infiltration as well as loss of lung architecture (Fig 3, *C*). We also detected a significant increase in the frequency and total number of IL-5– and IL-13–secreting T_H_2 cells and a trend toward an increase in IL-4–secreting T_H_2 cells in the lungs of *Cd11c*^*Cre*^*Map3k8*^*fl/ko*^ mice (Fig 3, *D*), concurrent with a significant increase in the production of HDM-specific IL-5 and IL-13 in the medLN cell cultures from *Cd11c*^*Cre*^*Map3k8*^*fl/ko*^ mice (Fig 3, *E*). As in *Map3k8*^*−*/*−*^ mice (Fig 1, *H*), we also observed a significant increase in total levels of circulating IgE in *Cd11c*^*Cre*^*Map3k8*^*fl/ko*^ mice (Fig 3, *F*). These results suggest that TPL-2 in CD11c^+^ DC negatively regulated airway inflammation after HDM challenge.

**Fig E4:**
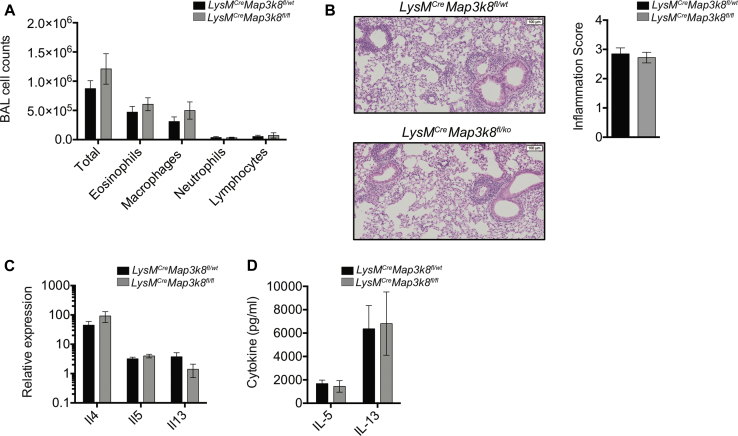
Role of TPL-2 function in LysM+ cells upon HDM sensitization and challenge. **A,** Differential counts in the BAL fluid of allergic *LysM*^*Cre*^ conditional KO mice. **B,** Hematoxylin and eosin–stained histology sections and inflammation scores from allergic lungs of *LysM*^*Cre*^ conditional KO mice. **C,** Lung expression of *Il4*, *Il5*, and *Il13* mRNA in allergic *LysM*^*Cre*^ conditional KO mice. **D,** HDM-specific IL-5 and IL-13 protein production as assessed by ELISA in the culture supernatants of medLN cells from allergic *LysM*^*Cre*^ conditional KO mice for 4 days. All experiments are representative of 3 independent experiments with 4 to 6 mice/genotype. *KO*, Knockout.

It was important to determine whether the enhanced allergic airway responses in *Map3k8*^*−*/*−*^ mice were attributable to DCs and not to any other CD11c-expressing cells, such as alveolar macrophages that would delete TPL-2 in *Cd11c*^*Cre*^*Map3k8*^*fl/ko*^ mice. To do this, we used a *LysM*^*Cre*^ driver mouse strain to delete TPL-2 in LysM-expressing cells, including alveolar macrophages. After HDM sensitization and challenge, *LysM*^*Cre*^*Map3k8*^*fl/ko*^ mice mounted a comparable airway inflammatory response to control mice, with similar airway infiltrates (see Fig E4, *A*, in this article's Online Repository at www.jacionline.org), comparable pulmonary inflammation (Fig E4, *B*), lung mRNA expression for type 2 cytokines (Fig E4, *C*), and HDM-specific cytokine secretion by restimulated medLN cells (Fig E4, *D*) upon HDM sensitization and airway challenge. These observations indicated that the exacerbated allergic airway inflammation observed in TPL-2–deficient mice was not due to a cell-intrinsic defect of TPL-2 in alveolar macrophages.

**Fig E5:**
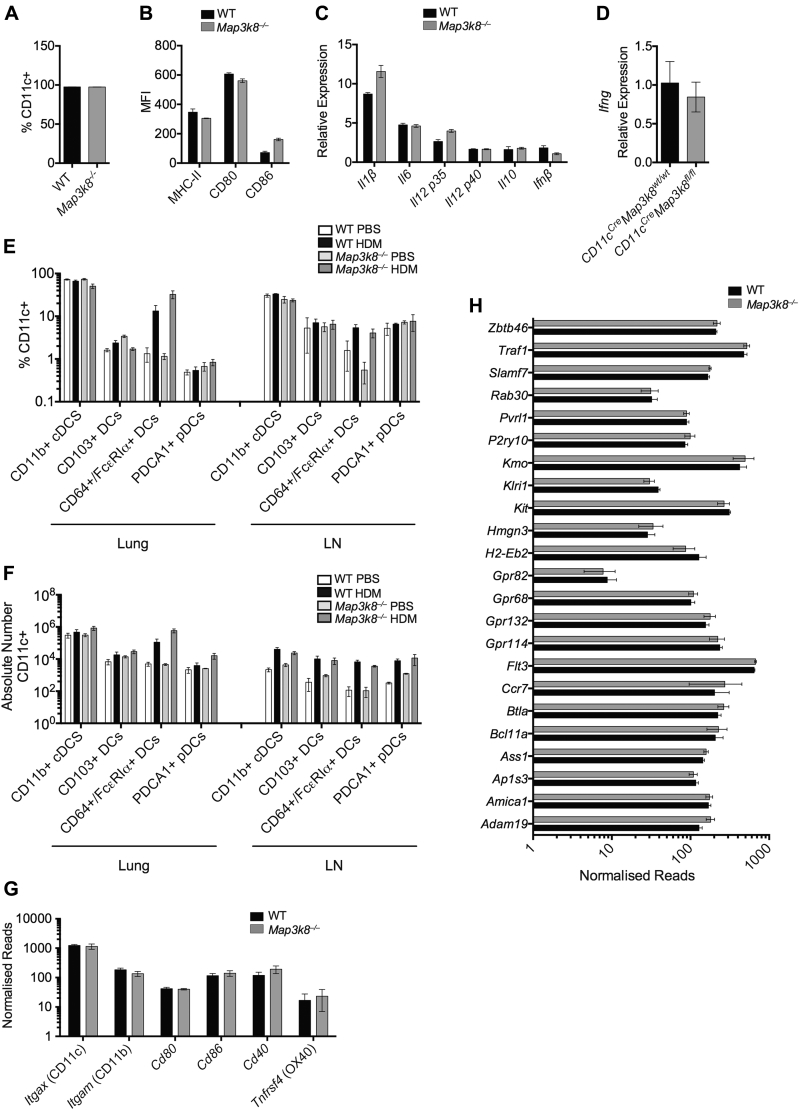
Characterization of WT and *Map3k8*^*−*/*−*^ DCs. **A,** Frequency of CD11c expression in HDM-stimulated WT and *Map3k8*^*−*/*−*^ BMDCs. **B,** MFI of antigen-presenting MHC-II molecule and costimulatory molecules CD80 and CD86 in HDM-stimulated WT and *Map3k8*^*−*/*−*^ BMDCs. **C,** Relative expression of previously identified targets (*Il1β*, *Il6*, *Il12 p35*, *Il12 p40*, *Il10*, and *Ifnβ*) of TPL-2 in HDM-stimulated WT and *Map3k8*^*−*/*−*^ BMDCs. **D,** Lung expression of *Ifng* mRNA in allergic *Cd11c*^*Cre*^ conditional KO mice. **E** and **F,** Frequency and total number of different subsets of CD11c^+^ DCs in the lung and medLNs of PBS- and HDM-challenged WT and *Map3k8*^*−*/*−*^ mice as assessed by intracellular cytokine staining. **G,** Normalized expression of DC-associated genes in *ex vivo* WT and *Map3k8*^*−*/*−*^ DCs. **H,** Normalized expression of core cDC signature genes different from macrophages in *ex vivo* WT and *Map3k8*^*−*/*−*^ DCs. All experiments are representative of 2 to 3 independent experiments with 3 to 5 mice/samples per genotype. *cDC*, Conventional DCs; *KO*, knockout; *MFI*, mean fluorescence intensity; *pDC*, plasmacytoid DCs.

**Fig 4:**
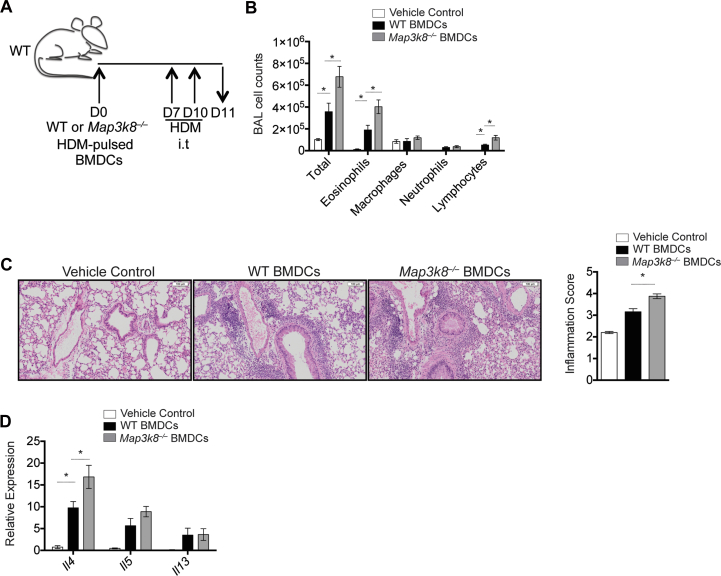
Adoptive transfer of *Map3k8*^*−*/*−*^ BMDCs in WT mice mediates increased allergic responses. **A,** Schematic representation of the adoptive transfer model for HDM-pulsed BMDCs and subsequent HDM challenge in naive WT mice. **B,** Differential counts in the BAL fluid of allergic WT mice adoptively transferred with vehicle control *(white bars)*, WT BMDCs *(black bars)*, or *Map3k8*^*−*/*−*^ BMDCs *(gray bars)*. **C,** Histology sections and inflammation scores from allergic lungs of WT mice adoptively transferred with WT, *Map3k8*^*−*/*−*^ BMDCs, or vehicle control depicting cellular infiltration. **D,** Lung expression of *Il4*, *Il5*, and *Il13* mRNA in allergic WT mice adoptively transferred with WT or *Map3k8*^*−*/*−*^ BMDCs or vehicle control. All experiments are representative of 2 to 3 independent experiments with 3 to 8 mice/genotype. *i.t*., Intratracheal. *P < .05 as assessed by Mann-Whitney test.

To further test whether DC-intrinsic TPL-2 was required to regulate airway inflammation, we used a well-established adoptive transfer system using HDM-pulsed bone marrow–derived dendritic cells (BMDCs).^1^ WT and *Map3k8*^*−*/*−*^ BMDCs (see Fig E5, *A* and *B*, in this article's Online Repository at www.jacionline.org) were pulsed overnight with HDM and then transferred via intratracheal administration into naive WT mice (Fig 4, *A*). In line with experiments with *Map3k8*^*−*/*−*^ mice and *Cd11c*^*Cre*^*Map3k8*^*fl/ko*^ mice, mice receiving a single adoptive transfer of *Map3k8*^*−*/*−*^ BMDCs followed by 2 intratracheal challenges displayed significantly increased infiltration of total cells, eosinophils, and lymphocytes in the BAL fluid compared with mice given HDM-pulsed WT BMDCs (Fig 4, *B*). Mice receiving unpulsed WT and *Map3k8*^*−*/*−*^ BMDCs failed to develop any inflammatory response (data not shown). Augmented pulmonary inflammation was also observed in mice given HDM-pulsed *Map3k8*^*−*/*−*^ BMDCs compared with WT BMDCs (Fig 4, *C*). Adoptive transfer of HDM-pulsed *Map3k8*^*−*/*−*^ BMDCs also resulted in higher levels of *ll4* and a trend toward an increase in *Il5* mRNA in the lung compared with control WT BMDCs (Fig 4, *D*). These observations support the hypothesis that DC-intrinsic TPL-2 regulated airway and tissue inflammation after HDM challenge.

### TPL-2 regulates the expression of *Ccl24* by dendritic cells

**Fig 5:**
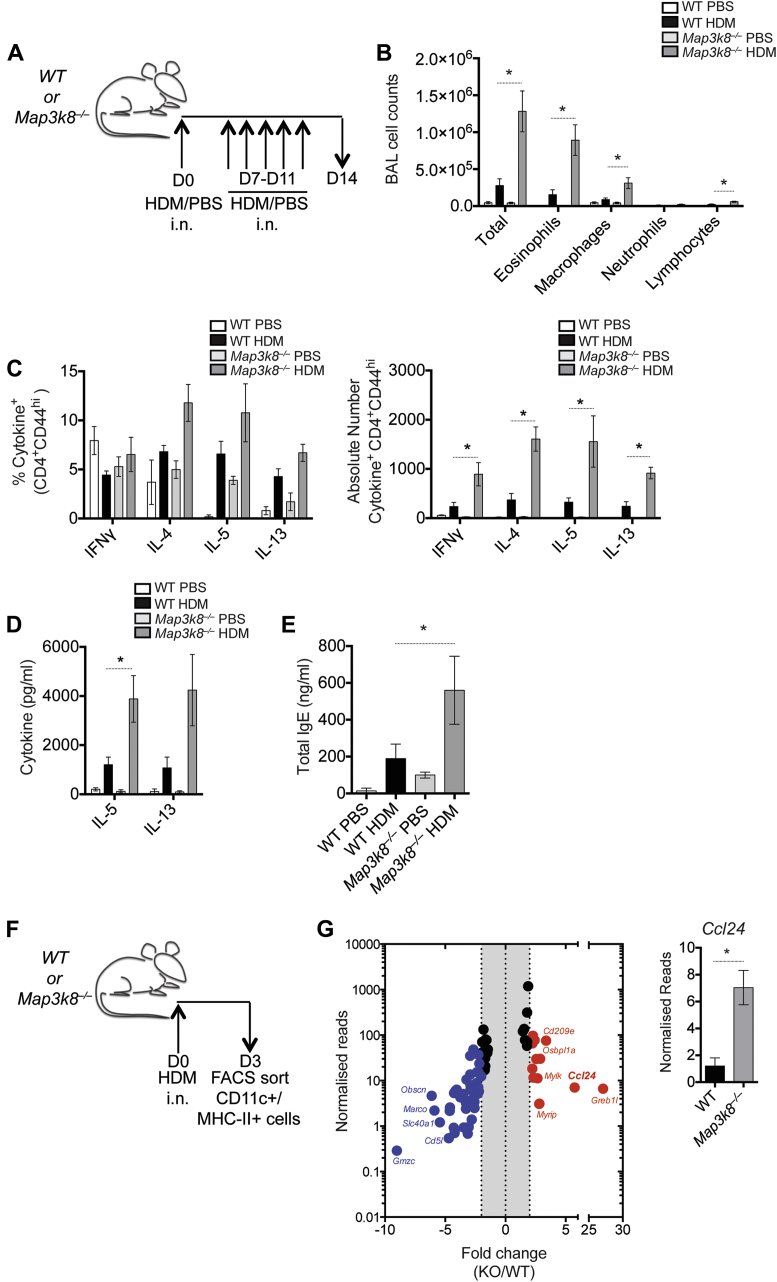
Expression profiling of WT and *Map3k8*^*−*/*−*^ DCs in an alum-independent model of HDM sensitization. **A,** Schematic representation of the alum-independent model of i.n. HDM sensitization and challenge in WT and *Map3k8*^*−*/*−*^ mice. **B,** Differential counts in the BAL fluid of PBS- and HDM-challenged WT and *Map3k8*^*−*/*−*^ mice. **C,** Frequency and total number of cytokine+/CD4+/CD44hi cells in the PBS- and HDM-challenged lungs of WT and *Map3k8*^*−*/*−*^ mice as assessed by intracellular cytokine staining. **D,** HDM-specific IL-5 and IL-13 protein production as assessed by ELISA in the cell culture supernatants of medLNs from PBS- and HDM-challenged WT and *Map3k8*^*−*/*−*^ mice for 4 days. **E,** Total IgE in the serum as assessed by ELISA from PBS- and HDM-challenged WT and *Map3k8*^*−*/*−*^ mice. **F,** Schematic representation for isolation of CD11c^+^/MHC-II^+^ DCs *ex vivo* after i.n. HDM sensitization. **G,** Plot depicting normalized gene expression levels of the *Map3k8*^*−*/*−*^ samples over the WT samples, reported as fold change. The *red data points* depict genes upregulated in the *Map3k8*^*−*/*−*^ samples over the WT samples, and the *blue data points* depict genes downregulated in the *Map3k8*^*−*/*−*^ samples over the WT samples. Gene expression of *Ccl24* plotted from the RNA sequencing analysis of CD11c^+^/MHC-II^+^ cells *ex vivo*. All experiments are representative of 2 independent experiments with 3 to 6 mice/genotype. The RNA sequencing analysis was performed on 3 independent samples/genotype. *FACS*, Fluorescence-activated cell sorting; *i.n*., intranasal; *KO*, knockout. *P < .05 as assessed by Mann-Whitney test.

Airway allergy induced by an intranasal (i.n.) HDM sensitization and i.n. challenge regimen (Fig 5, *A*) is strictly DC-dependent,^26^ more physiologically relevant, and does not require the use of aluminum hydroxide as an adjuvant. Using this model, we also observed a significant increase in total BAL cells and eosinophils in the BAL fluid of *Map3k8*^*−*/*−*^ mice, compared with WT controls (Fig 5, *B*). We observed an increase in the frequency of T_H_2 cells producing IL-4, IL-5, and IL-13, although this was not statistically significant, and a significant increase in the total number of IL-4–, IL-5–, and IL-13–secreting T_H_2 cells in the lungs of *Map3k8*^*−*/*−*^ mice compared with WT controls (Fig 5, *C*). In addition, *Map3k8*^*−*/*−*^ medLN cell cultures produced significantly more IL-5 and a trend toward more IL-13 after HDM restimulation, compared with WT medLN cell cultures (Fig 5, *D*). Serum levels of total IgE were also significantly elevated in *Map3k8*^*−*/*−*^ mice compared with WT controls (Fig 5, *E*). Together, these observations suggest that TPL-2–mediated control of allergic airway responses was independent of the route of antigen administration and independent of any alum adjuvant-associated effects.

To investigate how TPL-2 regulated DC function, we performed fluorescence-activated cell sorting of CD11c^+^MHC-II^+^ DCs to high purity (∼99%) from the local draining medLNs of intranasally sensitized WT and *Map3k8*^*−*/*−*^ mice (Fig 5, *F*) and performed RNA sequencing. Genomewide transcriptional analysis of purified DCs identified a number of characteristic DC-associated genes, which were comparable in both WT and *Map3k8*^*−*/*−*^ DCs (Fig E5, *H*).^27^ To identify transcriptional differences between WT and *Map3k8*^*−*/*−*^ DCs, mRNA expression in *Map3k8*^*−*/*−*^ DCs was compared to that in WT DCs and statistically significant differences in gene expression were identified using edgeR analysis (Fig 5, *G*). *Ccl24* (eotaxin-2), which functions as a potent eosinophil and granulocyte chemoattractant *in vivo* in both atopic and nonatopic patients^28^ and in animal models of airway allergy,^29^, ^30^ was approximately 5.7-fold upregulated in *Map3k8*^*−*/*−*^ DCs compared with WT DCs (Fig 5, *G*). Interestingly, the expression of Ccr3, the receptor for Ccl24, on T cells is regulated by IL-4 and IL-2^31^ and has previously been implicated in the preferential recruitment of T_H_2 cells.^32^ These data raised the possibility that DC TPL-2 regulated the recruitment of both T_H_2 cells and eosinophils by regulating the expression of Ccl24.

### TPL-2 restricts Ccl24 production to limit severe allergic airway inflammation

**Fig 6:**
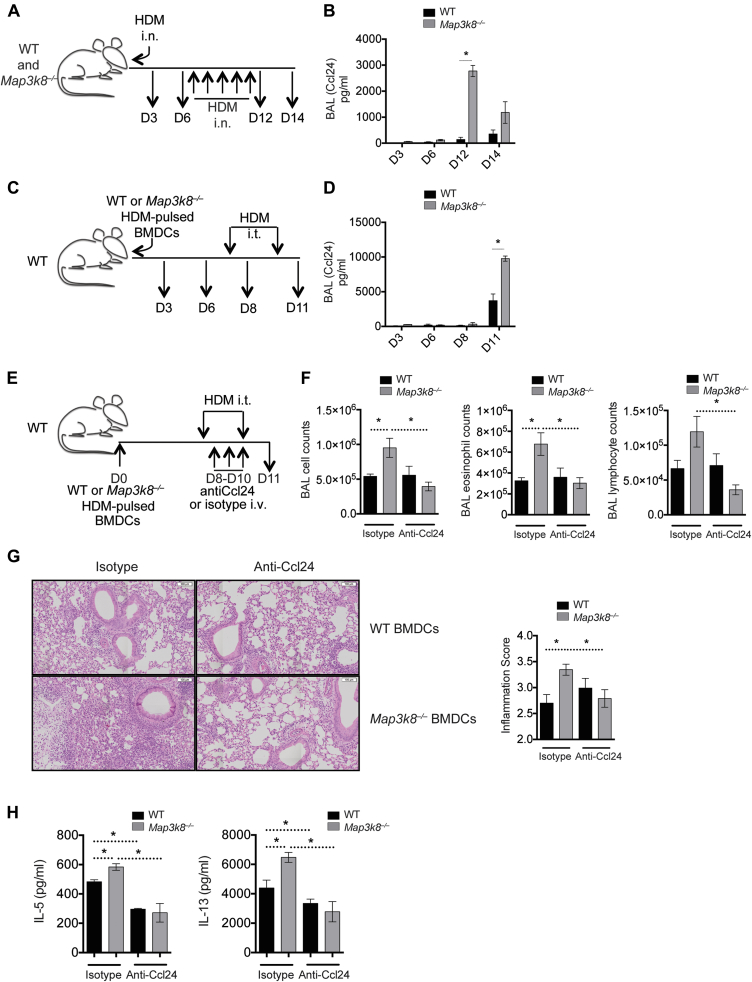
*Map3k8*^*−*/*−*^ BMDCs mediate increased eosinophilia and airway inflammation by regulating the production of Ccl24. **A,** Schematic representation of the time course for evaluating Ccl24 production in WT and *Map3k8*^*−*/*−*^ mice during the i.n. HDM sensitization and challenge model, as in Fig 5. **B,** Ccl24 protein in the BAL fluid of WT and *Map3k8*^*−*/*−*^ mice sensitized and challenged with HDM. **C,** Schematic representation of the time course for evaluating Ccl24 production in allergic mice following transfer of HDM-pulsed WT or *Map3k8*^*−*/*−*^ BMDCs. **D,** Ccl24 protein in the BAL fluid of allergic mice given HDM-pulsed WT and *Map3k8*^*−*/*−*^ BMDCs. **E,** Schematic representation of the setup for neutralizing Ccl24 in allergic mice using the adoptive transfer of HDM-pulsed WT or *Map3k8*^*−*/*−*^ BMDCs. **F,** Total number of all cells, eosinophils, and lymphocytes in the BAL fluid of allergic WT mice receiving with either HDM-pulsed WT or *Map3k8*^*−*/*−*^ BMDCs and given either anti-Ccl24 antibody or isotype control. **G,** Histology sections and inflammation scores from allergic lungs of WT mice adoptively transferred with WT or *Map3k8*^*−*/*−*^ BMDCs and given either anti-Ccl24 antibody or isotype control. **H,** HDM-specific IL-5 and IL-13 protein production as assessed by ELISA in the 4-day cell culture supernatants of medLNs from HDM-challenged WT mice given either WT or *Map3k8*^*−*/*−*^ BMDCs and either anti-Ccl24 antibody or isotype control. All experiments are representative of 2 to 3 independent experiments with 4 to 5 mice/genotype. *i.n*., Intransal; *i.t*., intratracheal; *i.v*., intravenous. *P < .05 as assessed by Mann-Whitney test.

Following the observation that *Map3k8*^*−*/*−*^ DCs had elevated *Ccl24* expression, we evaluated the production of Ccl24 protein by *Map3k8*^*−*/*−*^ mice after i.n. HDM sensitization and challenge (Fig 6, *A*). Ccl24 protein was barely detectable in BAL fluid after i.n. HDM sensitization (D3) in either WT or *Map3k8*^*−*/*−*^ mice (Fig 6, *B*). However, at days 12 and 14 after i.n. challenge, Ccl24 protein was clearly detected and significantly elevated on day 12 in the BAL fluid of *Map3k8*^*−*/*−*^ mice compared with WT mice (Fig 6, *B*). Thus, increased Ccl24 in *Map3k8*^*−*/*−*^ mice correlated with increased allergic responses (Fig 5 and Fig 6, *B*).

To determine whether *Map3k8*^*−*/*−*^ DCs were responsible for elevated Ccl24 *in vivo* and contributed to the severe allergic responses, we first evaluated the levels of Ccl24 in the BAL fluid of WT mice given either HDM-pulsed WT or HDM-pulsed *Map3k8*^*−*/*−*^ BMDCs (Fig 6, *C*). Similar to intact WT and *Map3k8*^*−*/*−*^ mice, we detected very low levels of Ccl24 in the BAL fluid at day 3 and day 6 after cell transfer (Fig 6, *D*). However, at day 11 after HDM challenge, we observed a significant increase in Ccl24 in mice given *Map3k8*^*−*/*−*^ BMDCs compared with mice given WT BMDCs (Fig 6, *D*). These results suggested that the absence of TPL-2 in DCs was responsible for increased Ccl24 in the BAL of HDM-challenged *Map3k8*^*−*/*−*^ mice, which correlated with severe airway allergy (Fig 4, *B*-*D*, and Fig 6, *D*).

To test whether elevated Ccl24 in mice receiving *Map3k8*^*−*/*−*^ BMDCs was responsible for severe airway inflammation, we adoptively transferred HDM-pulsed WT or *Map3k8*^*−*/*−*^ BMDCs into naive WT mice and neutralized Ccl24 using anti-Ccl24 antibodies (Fig 6, *E*). As demonstrated above, mice receiving *Map3k8*^*−*/*−*^ BMDCs and challenged with HDM had significantly higher airway inflammation with increased eosinophilia and lymphocyte recruitment (Fig 6, *F*). Ccl24 blockade had minimal effect on airway inflammation in mice given WT BMDCs; however, Ccl24 blockade completely prevented severe airway inflammation observed in mice given *Map3k8*^*−*/*−*^ BMDCs, compared with the isotype-treated control mice (Fig 6, *F*). Specifically, blocking Ccl24 reduced airway eosinophilia and lymphocyte recruitment in the BAL fluid of mice given *Map3k8*^*−*/*−*^ BMDCs, compared with isotype-treated mice (Fig 6, *F*). The increased pulmonary inflammation observed in mice given HDM-pulsed *Map3k8*^*−*/*−*^ BMDCs was also significantly reduced upon neutralization of Ccl24, compared with isotype-treated mice (Fig 6, *G*). In addition, allergic mice transplanted with *Map3k8*^*−*/*−*^ BMDCs had a significant increase in IL-5 and IL-13 production from restimulated HDM-specific medLN cultures compared with mice receiving WT BMDCs (Fig 6, *H*). This increased type 2 cytokine production in mice receiving *Map3k8*^*−*/*−*^ BMDCs was significantly reduced upon neutralization of Ccl24 compared with isotype-treated control mice (Fig 6, *H*). Surprisingly, neutralization of Ccl24 also led to a significant decrease in IL-5 and IL-13 production in mice given WT BMDCs compared with mice treated with isotype control (Fig 6, *H*). There was no significant difference between the type 2 cytokine production in mice given WT or *Map3k8*^*−*/*−*^ BMDCs along with Ccl24-neutralizing antibody (Fig 6, *H*). These observations demonstrated that severe airway allergy with elevated airway eosinophilia, lymphocyte infiltration, and lung inflammation in mice receiving *Map3k8*^*−*/*−*^ BMDCs was mediated by Ccl24. Furthermore, Ccl24 regulated HDM-specific type 2 cytokine production from LN cultures, suggesting that Ccl24 also regulated local lymphocyte recruitment. Collectively, these data identify a critical role for DC-intrinsic TPL-2 in preventing severe airway allergic responses to HDM by regulating Ccl24 expression.

## Discussion

In the present study, we identified that TPL-2 prevented severe airway allergic responses to HDM, mediated principally by a DC-intrinsic function of TPL-2. RNA sequencing of draining medLN DCs identified that TPL-2 critically regulated *Ccl24*.

Elevated Ccl24 protein was also observed in the BAL fluid of *Map3k8*^*−*/*−*^ mice after HDM challenge, suggesting that Ccl24 contributed to the severe airway disease in *Map3k8*^*−*/*−*^ mice. Consistent with this hypothesis, severe airway allergy induced by adoptive transfer of *Map3k8*^*−*/*−*^ DCs could be blocked by neutralization of Ccl24. Together, these results indicate that DC-intrinsic TPL-2 limits severe airway allergic responses by regulating the production of Ccl24.

The role of TPL-2 in regulating type-1 and T_H_17 inflammatory responses has been extensively investigated.^9^, ^10^, ^16^, ^17^ In contrast, only a single study has reported a role for TPL-2 in type 2 responses.^19^ In agreement with Watford et al,^19^ our study confirmed that TPL-2 inhibits type 2 responses. However, the present study, using a more physiologically relevant allergen (HDM) and in several models of airway allergy, clearly demonstrated that TPL-2 expression in T cells was not important in airway allergic responses. This contrasts with the conclusions of this earlier study in which *in vitro* experiments suggested that a T-cell–intrinsic role for TPL-2 was required to limit type 2 responses.^19^ Our results clearly demonstrated that TPL-2 was not required for T_H_2 differentiation *in vitro* and by restricting the deficiency of TPL-2 to T cells, using mixed BM chimeras or T-cell conditional knockout mice, it was evident that T-cell–intrinsic TPL-2 had no role in regulating type 2 responses *in vivo*.

A critical role of DCs in initiating and activating allergic type 2 responses has previously been demonstrated.^26^, ^33^ However, the molecular mechanisms by which DCs initiate type 2 response are incompletely understood. We did not observe any appreciable differences in the frequency or total number of different DC subsets (CD11b^+^ cDCs, CD103^+^ DCs, CD64^+^/FcεRIα^+^ inflammatory DCs, PDCA1^+^ plasmacytoid DCs) between WT and *Map3k8*^*−*/*−*^ mice at baseline or after HDM sensitization and challenge either in the lung or in medLNs, suggesting that TPL-2 did not regulate the development or recruitment of different DC subsets (Fig E5, *E* and *F*). However, we demonstrated that TPL-2 was an important regulator of DC-derived Ccl24, limiting the development of severe airway allergic responses to HDM. On the basis of experiments with *Cd11c*^*Cre*^*Map3k8*^*fl/ko*^ mice, *LysM*^*Cre*^*Map3k8*^*fl/fl*^ mice, and adoptive transfer experiments with BMDCs and using an intranasal sensitization and challenge system, we established that DC-intrinsic TPL-2 provided an important regulatory role to prevent the development of severe airway allergy. Strikingly, a single adoptive transfer of HDM-pulsed *Map3k8*^*−*/*−*^ DCs recapitulated many of the features of severe airway allergy, including increased airway and tissue inflammation, similar to HDM-challenged *Map3k8*^*−*/*−*^ mice.

Although the array of model systems used in this study converged upon a dominant DC-intrinsic role for TPL-2 in regulating airway and tissue inflammation, several differences also emerged between the model systems. These may be due to additional roles for TPL-2 in other cellular compartments, different routes of sensitization, frequency of airway challenges, and time of analysis. Furthermore, within the DC-specific systems, the role of different DC subsets and different CD11c-expressing cells may also contribute to different aspects of the allergic response. Additional comparative studies using both human and murine DC subsets from allergic and nonallergic individuals are needed to delineate the role of TPL-2 in regulating DC function.

To identify how DC-intrinsic TPL-2 regulated airway allergic responses, we compared the transcriptome of *ex vivo* CD11c^+^/MHC-II^+^ DCs from WT and *Map3k8*^*−*/*−*^ mice using a well-established model of DC-dependent HDM- mediated airway allergy.^22^ Increased allergic responses in *Map3k8*^*−*/*−*^ mice using this i.n. model indicated that TPL-2 inhibited airway allergic responses independent of the intraperitoneal priming or the potential impact of the aluminum hydroxide adjuvant. Previous studies have described a range of DC-expressed molecules, including CD40, CD80/86, and OX40, required to activate T_H_2 immunity.^34^, ^35^, ^36^, ^37^ We did not observe differential expression of any of these molecules between *Map3k8*^*−*/*−*^ and WT DCs (Fig E5, *B* and *G*). Our earlier work also established that TPL-2 regulates the expression of *Il12 p35, Il12 p40, Il10*, and *Ifnb* in TLR-activated DC.^10^ However, we did not observe differential regulation of any of these cytokines in *in vitro* HDM-pulsed *Map3k8*^*−*/*−*^ DCs (Fig E5, *C*). TPL-2 has been previously shown to regulate IFN-γ and type 1 responses.^15^, ^16^ However, following HDM-induced airway responses, we did not observe any significant difference in lung *Ifng*, between the allergic conditional knockout mice in the CD11cCre model (Fig E5, *D*), indicating that type 1 responses were intact in mice with TPL-2–deficient DCs. This was in contrast to the observation that allergic *Map3k8*^*−*/*−*^ mice had significantly reduced expression of *Ifng,* compared with allergic WT mice (Fig 1, *E*), suggesting CD11c^+^ cell independent control of *Ifng* expression in *Map3k8*^*−*/*−*^ mice following HDM sensitization and challenge. These differences suggest that DC-intrinsic TPL-2 regulates HDM-driven type 2 responses in a context-dependent manner, compared with type 1/TLR-mediated responses.

Ccl24 (eotaxin-2) is a chemoattractant for CCR3-expressing cells, predominantly eosinophils and T_H_2 lymphocytes,^32^, ^38^ and may contribute to airway remodeling.^39^ Hallmark features of type 2 human allergic asthma are circulating, airway, and tissue eosinophils accompanied by elevated T_H_2 lymphocytes, suggesting a mechanistic link between elevated DC expression of *Ccl24*, observed in this study, and elevated airway eosinophilia and type 2 immune responses. Although elevated *Ccl24* has been observed in IL-4–treated, type 2–promoting BMDCs and *ex vivo* DCs from allergic mice,^33^, ^40^ the functional relevance of DC-derived Ccl24 was not investigated in these studies. Murine studies presented here identified that *Map3k8*^*−*/*−*^ DCs were sufficient to invoke elevated levels of Ccl24 in the airways of mice following adoptive transfer, concomitant with increased eosinophilia and lymphocyte recruitment, highlighting the importance of Ccl24 in driving a severe allergic response.

The expression of CCR3 on T_H_2 cells^32^ provides a potential mechanistic explanation for how DC-derived Ccl24 could promote T_H_2-driven responses. Consistent with this, neutralizing Ccl24 dramatically reduced airway eosinophilia, lymphocyte infiltration, and lung inflammation in allergic mice given *Map3k8*^*−*/*−*^ BMDCs, highlighting a key role of Ccl24 in mediating severe airway allergy in *Map3k8*^*−*/*−*^ mice.

It will be important to determine whether TPL-2 also regulates *CCL24* in human DCs and whether the TPL-2 pathway is compromised in those with severe asthma. In support of this, sputum from patients with severe, treatment-refractory asthma had elevated levels of Ccl24, compared with the sputum of those with nonsevere asthma.^41^ Similarly, elevated expression of tissue Ccl24 has been observed in those with severe asthma, correlating with sputum eosinophilia, lower FEV_1_, and more asthma exacerbations.^42^ In addition, polymorphisms in the *CCL24* gene have been associated with the development of asthma.^43^ These data support the notion that elevated Ccl24 may contribute to severe airway allergy and maybe a useful biomarker to identify patients with severe asthma^44^ and supports the hypothesis that dysregulated TPL-2 in DCs may contribute to elevated Ccl24 in those with severe asthma; however, this needs to be tested.

Although we demonstrated that Ccl24 was an important mediator of severe allergic responses in mice, it was interesting to note that blockade of Ccl24 had little effect on the allergic airway responses in mice given WT BMDCs, most likely due to the lower levels of Ccl24 observed in the BAL fluid of mice given HDM-pulsed WT DCs or i.n. HDM (Fig 6). These data suggest that dysregulated Ccl24 may be responsible for the transition from moderate to severe asthma in mice and that other mediators, such as Ccl11 (eotaxin-1) and IL-5, may contribute to airway inflammation in moderate asthma that developed in WT mice. In support of this observation, Ccl24-deficient mice are capable of mounting low levels of airway eosinophilia after ovalbumin challenge.^30^ It is also possible that IL-5, Ccl11, and Ccl24 synergistically contribute to severe airway inflammation. Indeed, the coexpression of Ccl11 and Ccl24,^45^ or IL-5 and Ccl24,^46^ correlates with persistent airway eosinophilia in mice with severe airway allergy.

In conclusion, we have demonstrated that TPL-2 negatively regulates type 2 responses to HDM, preventing the development of severe airway allergic responses. Cell-intrinsic functions in T cells and B cells in airway allergic responses were ruled out, whereas TPL-2 expression in DCs was shown to be essential to regulate the expression of Ccl24 in the lungs after HDM challenge. Antibody-blocking experiments confirmed the importance of elevated Ccl24 in driving severe airway inflammation in *Map3k8*^*−*/*−*^ mice. Further study is required to determine whether TPL-2 regulates chemokine secretion, including Ccl24, in human cells and whether alterations in TPL-2 expression/signaling contribute to severe asthma. TPL-2 is widely considered as a promising anti-inflammatory drug target.^47^ However, the results in the present study suggest that TPL-2 inhibitors could have unwanted impacts on allergic comorbidities.

**Key message**

• TPL-2 functions in DCs to limit severe airway allergic responses to HDM by regulating Ccl24 production.

## Methods

### Animals

All mice were bred and maintained under specific pathogen-free conditions at the Francis Crick Institute. Strains used included WT C57BL/6, *Map3k8*^*−*/*−*^^E1^ on a C57BL/6 background, *Il4*^*GFP*^*Foxp3*^*RFP*^*10*^*BIT*^ (B6.*Il4*^*GFP*^ crossed with B6.*Foxp3*^*RFP*^.*10*^*BIT*^), *Map3k8*^*−*/*−*^*Il4*^*GFP*^*Foxp3*^*RFP*^*10*^*BIT*^ (*Map3k8*^*−*/*−*^ crossed with *Il4*^*GFP*^*Foxp3*^*RFP*^*10*^*BIT*^), *Cd4*^*Cre*^*Map3k8*^*flfl*^*R26*^*eYFP*^ (B6.*Cd4*^*Cre*^ *crossed with Map3k8*^*flfl*^ *and* B6*.R26*^*eYFP*^)*, Tcra*^*−/−*^ (B6.129S2-Tcra^tm1Mom^/J), *Mu*MT(*B10.129S2(B6)-Ighm*^*tm1Cng*^*/J*), C57BL/6 *Rag1*^*−*/*−*^, C57BL/6 *Rag2*^*−*/*−*^, *Cd11c*^*Cre*^*Map3k8*^*flfl*^*R26*^*eYFP*^ (B6*.Cd11c*^*Cre*^ *crossed with Map3k8*^*flfl*^ *and* B6*.R26*^*eYFP*^), and *LysM*^*Cre*^*Map3k8*^*flfl*^*R26*^*eYFP*^ (B6*.LysM*^*Cre*^ *crossed with Map3k8*^*flfl*^ *and* B6*.R26*^*eYFP*^). All animal experiments were carried out following UK Home Office regulations (project license 80/2506) and were approved by the Francis Crick Institute Ethical Review Panel.

### HDM-induced airway inflammation

As indicated, mice were sensitized twice via the intraperitoneal route with 100 μg (dry weight) of HDM (*Dermatophagoides pternoyssinus* extracts, Greer, Lenoir, NC), ovalbumin (10 μg, Sigma, St Louis, Mo), or Der p1 (10 μg; Greer) with Imject Alum (Thermoscientific, Waltham, Mass) diluted in PBS (1:3) solution. After sensitization, mice were challenged twice with 100 μg of HDM, Der p1, or ovalbumin, as indicated, and diluted intratracheally in PBS on days 21 and 24. All the parameters for airway allergy were measured 1 day after the last challenge.

### Intranasal sensitization and challenge model

Mice were sensitized intranasally with 100 μg of HDM diluted in PBS and subsequently challenged intranasally with 10 μg of HDM over a period of 5 days from day 7 to day 11. All the parameters for airway allergy were measured 3 days after the last challenge.

### BMDC culture and adoptive transfer

BM cells were cultured in the presence of 20 ng/mL GM-CSF (Peprotech, London, United Kingdom) for 10 days. For HDM pulsing, BMDCs were washed and cultured overnight with HDM (10 μg) and 5 ng/mL GM-CSF. The adoptive transfer model has been previously described.^E2^ Briefly, mature DCs were cultured overnight with 10 μg of HDM, washed with PBS, and transferred intratracheally into naive WT mice. After the initial transfer, mice were challenged with 100 μg of HDM diluted intratracheally in PBS on days 7 and 10. All the parameters for airway allergy were measured 1 day after the last challenge. Blocking antibodies diluted in PBS (Ccl24 antibodies [CCL24/eotaxin-2 MAb (Clone 106521, R&D Systems–Biotechne, Minneapolis, Minn) and CCL24/eotaxin-2 Affinity Purified Polyclonal Ab, R&D Systems–Biotechne]) were mixed and used at 0.03 mg/dose (monoclonal) and 0.006 mg/dose (polyclonal). Isotype controls rat IgG_2A_ (Clone 54447, R&D systems–Biotechne) and normal goat IgG (R&D systems–Biotechne) were mixed and used in similar ratios.

### Chimeric mice

C57BL/6 *Rag1*^*−*/*−*^ or C57BL/6 *Rag2*^*−*/*−*^ mice were irradiated with 900 RADs. For the T-cell–restricted chimeras, 2 × 10^6^ to 5 × 10^6^ mixed BM cells of *Tcra*^−/−^ and *Il4*^*GFP*^*Foxp3*^*RFP*^*10*^*BIT*^ or *Map3k8*^*−/−*^ *Il4*^*GFP*^*Foxp3*^*RFP*^*10*^*BIT*^ (ratio 80:20) were injected intravenously. For the B-cell–restricted chimeras, 2 × 10^6^ to 5 × 10^6^ mixed BM cells of *Mu*MT and *Il4*^*GFP*^*Foxp3*^*RFP*^*10*^*BIT*^ or *Map3k8*^*−/−*^ *Il4*^*GFP*^*Foxp3*^*RFP*^*10*^*BIT*^ (ratio 80:20) were injected intravenously. After 8 weeks, mixed BM chimeric mice were sensitized and challenged with HDM, as described above.

### Airway resistance measurements

Baseline and methacholine-induced airway hyperreactivity measurements of conscious, restrained mice were obtained using a Buxco FinePointe System for noninvasive airway measurement (Buxco Research Systems, Wilmington, NC) according to the manufacturer's instructions. Mice were exposed to aerosolised methacholine (Sigma), administered from 3 to 50 mg/mL for a 5-minute period (30 seconds dosing, 2 minutes recording, and 2.5 minutes recovery), with airway resistance measurements made during the 2 minutes of recording.

### BAL fluid preparation and differential cell counts

One day after the last HDM challenge, mice were culled and BAL fluid collected using 1.5 mL of PBS for each mouse. BAL fluid was centrifuged at 300*g* for 10 minutes at 4°C and cells resuspended in equal volumes of complete Iscove's modified Dulbecco's medium (IMDM) (Sigma) (with 10% FCS, 1% 100 U/mL penicillin and 100 μg/mL streptomycin [Gibco, Waltham, Mass], 8 mM l-glutamine [Gibco], and 0.05 mM 2-mercaptoethanol [Gibco]). The BAL fluid was stored at −80°C before analysis. The total number of BAL cells was counted and differential cell counts performed on cytospin preparations stained with Giemsa stain, modified (Sigma).

### Histology and inflammation scoring

Excised lungs were fixed in 10% neutral buffered formalin overnight and washed in 75% ethanol. The tissues were embedded in paraffin, and lung sections were stained with hematoxylin and eosin stain. Stained slides were scanned with a VS120-SL slide scanner (Olympus, Tokyo, Japan) and images were analyzed with the OlyVIA image viewer (Olympus). Inflammation scoring was performed as previously described.^E3^

### Isolation of lung and medLN cells

Mice were culled, bled, and lungs and medLNs collected. Lungs were homogenized and lymphocytes enriched by density gradient centrifugation using 40% Percoll (GE Healthcare, Little Chalfont, United Kingdom). Homogenized medLN cells were cultured at a density of 200,000 cells/well in 200 μL in 96-well round-bottom plates (Sigma) for 4 days, with 10 μg/mL HDM.

### Isolation of DCs

For the isolation of *ex vivo* DCs, medLNs from HDM-sensitized mice were homogenized and separated on a density gradient. To do this, cells were resuspended in a 15% w/v solution of OptiPrep (Sigma) and overlayed with the diluent solution (0.88% w/v sodium chloride, 1 mM ethylene-diamine-tetra-acetic acid, 0.5% w/v BSA, 10 mM Hepes-NaOH, pH 7.4) and IMDM. Samples were centrifuged at 600*g* for 15 minutes at room temperature and the enriched DC layer was collected from the top of the gradient for downstream analysis.

### Cell sorting and flow cytometry

Cell sorting was performed using a FACS Aria II (BD Biosciences, San Jose, Calif) cell sorter. For sorting, cell suspensions were stained for 20 minutes with antibodies in PBS with 2% FCS and then diluted in phenol-red free IMDM (Gibco) (with 1% FCS, 2 mM EDTA [Invitrogen], 100 U/mL penicillin and 100 μg/mL streptomycin [Gibco], 8 mM l-glutamine [Gibco], and 0.05 mM 2-mercaptoethanol [Gibco]). Propidium iodide or LIVE/DEAD fixable blue dead cell stain (Life Technologies) was used to determine cell viability. Cells were stained for surface antigens by incubation with antibodies in PBS with 2% FCS (20 minutes at 4°C). Intracellular cytokine staining was performed after 6 hours of restimulation with 50 ng/mL phorbol 12-myristate 13-acetate (Promega, Madison, Wis) and 1 μg/mL ionomycin (Sigma) and BD Golgi Stop and BD Golgi Plug (diluted 1:1000, BD Biosciences). After staining for surface antigens, cells were fixed and permeabilized (Fixation/Permeabilization diluent; eBioscience, San Diego, Calif), before incubation with cytokine antibodies in permeabilization buffer (eBioscience) for 20 minutes at 4°C. Cells were analyzed using a BD LSRII flow cytometer (BD Biosciences) and data processed using FlowJo software (Version X 10.0.7r2, Treestar Inc, Ashland, Ore). Antibodies used were purchased from eBioscience, Biolegend (San Diego, Calif), or BD Pharmingen (San Diego, Calif). They include CD3 (17A2), CD4 (RM4-5, GK1.5), CD11b (M1/70), CD11c (N418), CD19 (6D5, eBio1D3), CD25 (PC61), CD44 (IM7), CD45 (30-F11), CD49b (DX5), CD64 (X54-5/7.1), CD69 (H1.2F3), CD80 (16-10A1), CD86 (GL1), CD103 (2E7), CD197 (4B12), CD278 (C39.4A), F4/80 (BM8), FceRI (MAR-1), GATA-3 (L50-823), Gr-1 (RB6-8C5) (IFN-γ) (XMG1.2), I-A/I-E (M5/114.15.2), IL-4 (11B11), IL-5 (TRFK5), IL-13 (eBio13A), KLRG1 (2F1), Ly6G (1A8), NK1.1 (PK136), PDCA1 (927), SiglecF (E50-2440), TCR β chain (H57-597), TCRγδ (GL3), and Ter119 (TER-119). Lineage panel for the group 2 innate lymphoid cell staining was defined as CD4^+^/CD8^+^/CD3^+^/CD19^+^/TCRβ^+^/TCRγδ^+^/NK1.1^+^/CD49b^+^/CD11c^+^/CD11b^+^/Gr-1^+^/Ter119^+^. Staining was performed in the presence of FcR Blocking Reagent (Miltenyi Biotec, Bergisch Gladbach, Germany).

### T_H_2 polarization

Naive CD4^+^ T cells were sorted from spleens of WT or *Map3k8*^*−*/*−*^ mice as CD4^+^TCRβ^+^CD44^−^CD25^−^PI^−^. They were cultured for 6 days *in vitro* with 10 ng/mL IL-4 (R&D Systems–Biotechne, Minneapolis, Minn), 5 ng/mL IL-2 (R&D), 10 μg/mL anti–IFN-γ (XMG1.2, BioXcell, West Lebanon, NH), and CD3 (0.1-4.0 μg) (145-2C11, BioXcell) and CD28 (10 μg/mL) (37.51, BioXcell) in IMDM with 10% FCS (and other components as listed above).

### RT-PCR, ELISA, and Western blotting

Tissue samples were frozen in RNA*later* (Sigma) and homogenized in QIAzol (Qiagen, Hilden, Germany). Cell pellets were lysed in Buffer RLT (Qiagen). Total RNA was isolated as per manufacturer's protocol. Total RNA (100 ng-1 μg) was reverse transcribed using the QuantiTect Reverse Transcription Kit (Qiagen). cDNA produced was used for real-time quantitative PCR with Power SyBrGreen (Applied Biosystems, Foster City, Calif). The expression levels of different genes (*Ifng* [forward: ACAGCCAGATTATCTCTTTCTACCTCAG; reverse: CCTTTTTCGCCTTGCTGTTG], *Il4* [forward: ACGAGGTCACAGGAGAAGGGA; reverse: AGCCCTACAGACGAGCTCACTC], *Il5* [forward: TGACAAGCAATGAGACGATGAGGG; reverse: ACCCCCACGGACAGTTTGATTC], *Il13* [forward: CCTCTGACCCTTAAGGAGCTTAT; reverse: CGTTGCACAGGGGAGTCTT], *Il1β* [forward: GGAGAACCAAGCAACGACAAAATA; reverse: TGGGGAACTCTGCAGACTCAAAC], *Il6* [forward: TCCAGTTGCCTTCTTGGGAC; reverse: GTGTAATTAAGCCTCCGACTTG], *Il12p35* [forward: GAGGACTTGAAGATGTACA; reverse: TTCTACTTGGAGGAGGGC], *Il12 p40* [forward: GACCCTGCCGATTGAACTGGC; reverse: CAACGTTGCATCCTAGGATCG], *Il10* [forward: ATGCTGCCTGCTCTTACTGACTG; reverse: CCCAAGTAACCCTTAAAGTCCTGC], and *Ifnβ* [forward: GCACTGGGTGGAAGTAGACT; reverse: AGTGGAGAGCAGTTGAGGACA]) were normalized to hypoxanthine-guanine phosphoribosyl transferase expression and expressed as fold change relative to naive or PBS-treated WT samples. Ccl24, IL-5, and IL-13 were measured using DuoSet ELISA kits, according to the manufacturer's instructions (R&D Systems–Biotechne). Total IgE ELISA was performed by coating with purified rat antimouse IgE (R35-72, BD Pharmingen) at 2 μg/mL overnight, followed by overnight incubation with serum and standard (purified mouse IgE, κ isotype standard, BD Pharmingen), and detection with Biotin rat antimouse IgE at 1 μg/mL (R35-118, BD Pharmingen), Streptavidin HRP (BD Pharmingen), and TMB ELISA substrate (eBioscience). For immunoblotting, cell lysates were normalized to equal total protein content and resolved on 10% Criterion TGX Gels (Biorad, Hercules, Calif). Separated proteins were transferred onto Trans-Blot Turbo PVDF transfer (Biorad) membranes. Specific bound antibodies were visualized by chemiluminescence (Immobilon; Merck Millipore, Darmstadt, Germany).

### RNA Sequencing

Raw Illumina reads were analyzed as follows. First, data quality was analyzed using FastQC (www.bioinformatics.babraham.ac.uk/projects/fastqc). Low-quality bases were trimmed using Trimmomatic.^E4^ Read pairs that passed the trimming quality filters were then aligned to mm10 (Ensembl version 75) using Tophat2.^E5^ Counts were determined using htseq_count.^E6^ Normalization and statistical analysis were performed using edger.^E7^ Statistically significant genes with a false discovery rate (FDR) of less than 0.05 are reported.

### Statistical analysis

Data sets were compared by using the Mann-Whitney test using GraphPad Prism (V.5.0). Differences were considered significant at a *P* value of .05 or less.
